# Application of Zinc Ferrite Nanoparticles for the Magnetic Removal of Algae That Bind Cadmium

**DOI:** 10.3390/nano16060361

**Published:** 2026-03-16

**Authors:** Péter Koska, Tímea Fóris, Kitti Gráczer, Ágnes Mária Állné Ilosvai, Ferenc Kristály, Lajos Daróczi, László Vanyorek, Béla Viskolcz

**Affiliations:** 1 Institute of Chemistry, University of Miskolc, Miskolc-Egyetemváros, 3515 Miskolc, Hungary; peter.zoltan.koska@uni-miskolc.hu (P.K.); timea.foris1@uni-miskolc.hu (T.F.); kitti.graczer@uni-miskolc.hu (K.G.); maria.agnes.ilosvai@uni-miskolc.hu (Á.M.Á.I.); 2 Higher Education and Industrial Cooperation Centre, University of Miskolc, 3515 Miskolc, Hungary; 3 Institute of Mineralogy and Geology, University of Miskolc, Miskolc-Egyetemváros, 3515 Miskolc, Hungary; ferenc.kristaly@uni-miskolc.hu; 4 Department of Solid State Physics, University of Debrecen, P.O. Box 2, 4010 Debrecen, Hungary; lajos.daroczi@science.unideb.hu

**Keywords:** *Chlorella vulgaris*, zinc ferrite, cadmium adsorption, magnetic separation

## Abstract

The removal of cadmium from contaminated water remains a critical challenge due to its high toxicity, persistence, and limited treatability at low concentrations. In this study, we propose a novel algal–nanoparticle system that integrates cadmium adsorption by *Chlorella vulgaris* with zinc ferrite (ZnFe_2_O_4_) nanoparticle-assisted sedimentation, with the aim of addressing a significant operational challenge in algal remediation. The microalgal biomass demonstrated the capacity to remove cadmium with efficiencies exceeding 90%, facilitated by adsorption through surface functional groups. The incorporation of ZnFe_2_O_4_ nanoparticles promoted the formation of dense, magnetically responsive aggregates, significantly accelerating biomass settling without the necessity for additional chemical flocculants. The strategy’s efficacy is evidenced by its enhancement of metal removal and solid–liquid separation processes, which renders it a potentially scalable and environmentally sustainable approach for the treatment of cadmium-contaminated wastewater. The strategy holds relevance for effluents derived from mining, electroplating, fertilizer production and battery manufacturing.

## 1. Introduction

Water contamination by heavy metals is one of the most significant environmental challenges of the modern era [1,2]. The continuous discharge of toxic pollutants into aquatic ecosystems has been attributed to several factors, including rapid industrialization, urban expansion, and intensified agricultural practices [1,2]. Among these contaminants, cadmium (Cd^2+^) has attracted particular attention due to its elemental persistence, high toxicity, and propensity for bioaccumulation in food webs [3,4,5,6]. Cadmium contamination of natural waters can originate from multiple industrial and agricultural activities, including mining and smelting, often as a by-product of Zn/Pb/Cu processing, electroplating, and the manufacture of cadmium-containing products [3,6,7]. In the context of agriculture, the utilization of phosphate fertilizers has been demonstrated to result in the introduction of cadmium (Cd) into the soil matrix. This Cd can subsequently mobilize within the soil profile, disseminating into surface waters and groundwater [3,5]. Additional contributions arise from cadmium use in pigments, plastic stabilizers, and nickel–cadmium batteries, as well as from electronic waste streams and recycling activities that disseminate Cd into the surrounding environments [6,7,8]. It is well-documented that exposure to cadmium poses significant risks to human health. These include renal toxicity, skeletal damage and carcinogenic effects. Furthermore, exposure to cadmium can also impair aquatic biota and destabilize ecosystem functioning [4,5]. Consequently, the removal of cadmium from wastewater streams remains an urgent priority in the fields of environmental science and engineering [1,2].

The efficacy of conventional wastewater treatment methodologies, encompassing chemical precipitation, ion exchange, membrane-based separations (e.g., reverse osmosis), and electrochemical approaches, has exhibited inconsistency in the context of cadmium remediation. Nevertheless, these methodologies frequently necessitate substantial operational expenses, result in sludge generation or the generation of secondary waste streams, demand intricate infrastructure, and/or exhibit diminished efficiency at low contaminant concentrations [1,2]. In response to these challenges, there has been an increasing focus on strategies that draw inspiration from biological systems and utilize nanotechnology to enhance both the efficiency and sustainability of these processes [1,2]. One such approach involves the coupling of microalgal adsorption with magnetically assisted separation, utilizing zinc ferrite (ZnFe_2_O_4_) nanoparticles to expedite the harvesting of cadmium-adsorbed algal biomass [9,10,11,12,13,14].

This combined strategy offers complementary advantages. *Chlorella vulgaris*, a green microalga, has been found to exhibit a remarkable capacity to adsorb cadmium through its cell surface functional groups, achieving up to 95% removal efficiency under optimized conditions [10,11]. Concurrently, the application of zinc ferrite nanoparticles expedites the sedimentation process, thereby circumventing a pivotal operational impediment in microalgal wastewater treatment, namely the protracted and energy-intensive harvesting of microalgal biomass [12]. The integration of adsorption and magnetic nanoparticle-assisted settling in this method ensures the expeditious separation of contaminated biomass from treated water. Furthermore, it facilitates the potential recovery and recycling of both metals and biological material [13,14]. Zinc ferrite has been the focus of investigation as a magnetically responsive material compatible with aqueous environmental applications. It has been demonstrated as a magnetic flocculant for microalgae harvesting when appropriately functionalized [14,15]. In a broader sense, the principles of magnetic harvesting have been demonstrated to support process intensification and may therefore enable downstream options for the recovery of resources. Such options include the reuse of nanoparticles and the safer handling of biomasses that are enriched with metals [13,15]. Beyond biomass harvesting, ZnFe_2_O_4_-based materials have also been widely explored in environmental remediation as photocatalysts and adsorbents for the removal of dyes, antibiotics, phosphate, and heavy metals from water [16,17,18,19,20]. Magnetically recoverable ZnFe_2_O_4_@g-C_3_N_4_ heterostructures have been applied to visible light-driven methylene blue degradation [16], while ZnFe_2_O_4_ systems have also been reported for the photocatalytic removal of tetracycline and ciprofloxacin from aqueous media [17,18]. In addition, hierarchical ZnO/ZnFe_2_O_4_ yolk shell adsorbents have been proposed for phosphate recovery and adsorption of organic pollutants from simulated wastewater, and Mg-doped ZnFe_2_O_4_ powders have shown rapid simultaneous uptake of Pb^2+^, Cu^2+^, and Cd^2+^ ions [19,20].

From an application perspective, cadmium-bearing effluents are recurrent in sectors such as mining and metallurgy, electroplating, pigment and polymer manufacturing (via cadmium pigments and stabilizers), and battery-related industries. Furthermore, the generation and recycling of electronic waste has the potential to disseminate Cd into compartments that are relevant to water systems [3,6,7,8]. Consequently, the utilization of ZnFe_2_O_4_ nanoparticle-assisted separation of Cd^2+^-adsorbed *Chlorella* biomass emerges as a promising technology for scalable remediation, aligning with current priorities in sustainable water management and green nanotechnology [1,2,11,12,15]. The novelty of the present work lies in the application of ZnFe_2_O_4_ nanoparticles for the magnetic recovery of Cd^2+^-adsorbed *Chlorella* biomass in a water treatment context, together with the pH-dependent electrokinetic interpretation of nanoparticle–cell interactions. Moreover, the ZnFe_2_O_4_ exhibits significant photocatalytic activity, which can be utilized for the degradation of microalgal cell walls to facilitate intracellular oil extraction, as demonstrated by Seo et al. [14]. For the downstream processing of cadmium-laden algae, photocatalytic cell lysis mediated by zinc ferrite offers a promising route for biomass valorization. Consequently, in this system, zinc ferrite serves not only as a magnetic agent for harvesting but also as a potential photocatalyst for subsequent biorefinery steps.

## 2. Materials and Methods

### 2.1. Materials

Zinc ferrite (ZnFe_2_O_4_) magnetic nanoparticles were synthesized from Zn(NO_3_)_2_∙6H_2_O, MW:297.47 g/mol (Thermo Fisher GmbH, D-76870 Kandel, Germany) and iron(III) nitrate nonahydrate, Fe(NO_3_)_3_∙9H_2_O (VWR International, B-3001 Leuven, Belgium). Ethylene glycol, HOCH_2_CH_2_OH (VWR Int. Ltd., F-94126 Fontenay-sous-Bois, France), was used as solvent. Monoethanolamine (MEA), NH_2_CH_2_OH (Merck KGaA, D-64271 Darmstadt, Germany) and sodium acetate, CH_3_COONa (ThermoFisher GmbH, D-76870 Kandel, Germany) were applied for coprecipitation of the nanoparticles. Potassium nitrate, KNO_3_, magnesium sulfate heptahydrate, MgSO_4_∙7H_2_O, potassium dihydrogen phosphate, KH_2_PO_4_, iron(II) sulfate heptahydrate, FeSO_4_∙7H_2_O, calcium chloride dihydrate, CaCl_2_∙2H_2_O, ethylenediaminetetraacetic acid disodium salt, boric acid, H_3_BO_3_, zinc sulfate heptahydrate ZnSO_4_∙7H_2_O, manganese(II) chloride tetrahydrate, MnCl_2_∙4H_2_O, sodium molybdate dihydrate, Na_2_MoO_4_∙2H_2_O, copper(II) sulfate pentahydrate CuSO_4_∙5H_2_O and citric acid (Merck KGaA, D-64271 Darmstadt, Germany) were used for microalgal cultivation.

### 2.2. Synthesis of the Zinc Ferrite Nanoparticles

Two types of synthesis methods were used to prepare amine-functionalized zinc ferrite in order to produce magnetic nanoparticles with different morphologies and size distributions. The synthesis methods for the ZnFe_2_O_4_ ST. and ZnFe_2_O_4_ Refl. samples differ primarily in the pressure applied during the process. The ZnFe_2_O_4_ ST. sample was prepared under solvothermal conditions in an autoclave at 200 °C under autogenous pressure. In contrast, the ZnFe_2_O_4_ Refl. sample was synthesized at atmospheric pressure, also at 200 °C, in a round-bottom flask under constant stirring and reflux. In both synthesis procedures, identical precursors and reactants were used in the same quantities.

The synthesis of ZnFe_2_O_4_ ST. sample under pressure was carried out in Teflon lined autoclave (volume: 150 mL), at 200 °C, 12 h. In the first step, zinc(II) nitrate hexahydrate (18 mmol) and iron(III) nitrate nonahydrate (36 mmol) were solved in 100 mL ethylene glycol (EG). Sodium acetate (90 mmol) was solved in the solution of the metal precursors and stirred at room temperature until dissolution, followed by the addition of 60 mL (0.9921 mol) monoethanol amine. The solution was transferred to an autoclave, and after it was heated at 200 °C, 12 h. After the synthesis, the solid phase was separated by magnet from the ethylene glycol phase, and it was washed by distilled water. The magnetic zinc ferrite (ZnFe_2_O_4_ ST.) was dried at 90 °C overnight.

The ZnFe_2_O_4_ Refl. sample was prepared at atmospheric pressure by refluxing and heating for 12 h. The amounts of reactants, the reaction temperature and the synthesis time were the same as for solvothermal synthesis. The difference was that the synthesis was carried out in a round-bottom flask under reflux with continuous stirring. After synthesis, the sample was washed and dried as described above.

### 2.3. Cadmium Adsorption Tests

#### 2.3.1. Microalgal Strain, Maintenance, and Cultivation

*Chlorella vulgaris* CCAP 211/109 (non-axenic isolate) was obtained from the Collection of Algae and Protozoa (CCAP; SAMS Limited, Dunbeg, Oban PA37 1QA, Scotland, UK). Stock cultures were maintained in liquid medium and subcultured every 2–3 months. Between transfers, cultures were stored at 4 °C to preserve viability.

Microalgal cultivation was performed using a modified Endo medium, previously reported for heterotrophic growth of *Chlorella vulgaris* with the aim of achieving high biomass concentrations [21]. The medium composition was as follows (per liter): KNO_3_, 3.0 g; KH_2_PO_4_, 1.2 g; MgSO_4_·7H_2_O, 1.2 g; citric acid, 0.3 g; FeSO_4_·7H_2_O, 0.016 g; CaCl_2_·2H_2_O, 0.105 g. After dissolution of the macronutrients, 1 mL L^−1^ of a trace element stock solution was added. The trace element solution contained (per liter): EDTA-Na_2_, 2.1 g; H_3_BO_3_, 2.86 g; ZnSO_4_·7H_2_O, 0.222 g; MnCl_2_·4H_2_O, 1.81 g; Na_2_MoO_4_·2H_2_O, 0.021 g; and CuSO_4_·5H_2_O, 0.07 g. The pH of the culture medium was adjusted to 6.5 ± 0.2 prior to inoculation.

Cultivation experiments were conducted in an airlift photobioreactor system consisting of twelve cylindrical glass reactors (nominal volume: 500 mL), supplied by a Hailea V20 diaphragm air compressor and illuminated by four Sylvania LED lamps. Each reactor was operated with a working volume of 400 mL to prevent foaming during aeration. The LED light sources provided an individual luminous flux of approximately 3000 lumens.

Aeration was supplied continuously at a total airflow rate of 20 L min^−1^, corresponding to a specific aeration rate of 4.16 L min^−1^ L^−1^ of culture volume. Uniform aeration among reactors was ensured by symmetric distribution of the airflow, with identical tubing lengths and an equal number of branch points between the compressor and each reactor.

Cultures were inoculated at 2% (*v*/*v*) using an actively growing inoculum with an initial cell density of approximately 1 × 10^9^ cells mL^−1^. Cultivation was carried out under continuous aeration and illumination (24 h d^−1^). The pH of the cultures was monitored at least once every 24 h using a VOLTCRAFT PHT-700 pH meter (Conrad Electronic SE, D-92242 Hirschau, Germany). Microalgal growth was followed by measuring optical density at 680 nm using a UV-6300 double-beam spectrophotometer (VWR International, Radnor, PA 19087, USA).

#### 2.3.2. Cadmium Exposure Experiments and Analytical Determination

Cadmium exposure experiments were initiated by the direct addition of a cadmium stock solution to the bioreactors immediately prior to inoculation. Final nominal cadmium concentrations in the cultures were adjusted to 8.6, 20.9, and 45.6 mg L^−1^. Each concentration was investigated in triplicate independent reactors.

Samples were collected at defined time points following cadmium addition (0.5, 16, 24, 48, 72, 120, and 148 h). At each sampling point, culture samples were divided into two aliquots. One aliquot was used for the determination of optical density at 680 nm (OD_680_) to estimate cell density. The second aliquot was centrifuged at 9000× *g* for 5 min to obtain cell-free supernatants for dissolved cadmium analysis.

For elemental analysis, 1.0 mL of the cell-free supernatant was transferred into a 15 mL polypropylene centrifuge tube (Falcon type, Corning Life Sciences, Durham, NC 27712, USA). Subsequently, 1.0 mL of concentrated hydrochloric acid was added for sample acidification, and the volume was adjusted to 10 mL with deionized water. Prepared samples were stored at ambient temperature until analysis.

Dissolved cadmium concentrations were determined using an inductively coupled plasma optical emission spectrometer (ICP-OES)

### 2.4. Magnetic Separation Tests of the Cadmium Bonded Algae

The experiments measured the time and efficiency of magnetic separation of cadmium-adsorbed algal biomass at different concentrations of algae. A concentrated *Chlorella* culture containing 4.2 g L^−1^ of dry biomass was diluted. Different concentrations of algal suspension were used, 3.6 g L^−1^ (=2.0 ± 0.1 OD680), 2.7 g L^−1^ (=1.5 ± 0.1 OD680), 1.8 g L^−1^ (=1.0 ± 0.1 OD680), 0.9 g L^−1^ (=0.5 ± 0.1 OD680), while the concentration of magnetic nanoparticles was fixed at 0.511 g L^−1^. A 100 cm^3^ algal suspension of different concentrations was mixed with a cadmium solution of 45 mg L^−1^ and air bubbling for 30 min. The algal biomass was treated with amine (-NH_2_)-functionalized zinc ferrite nanoparticles (0.511 g L^−1^) (either ZnFe_2_O_4_ Refl. or ZnFe_2_O_4_ ST.) at the same concentration for 10 min using the air bubbling compressor. Accordingly, the nanoparticle-to-biomass mass ratios were 0.142:1, 0.189:1, 0.284:1, and 0.568:1. The pH was adjusted to 6.5 using phosphate buffer, and the temperature was maintained at 24 °C.

To ascertain the efficiency with which nanoparticles bind to the surface of cadmium-adsorbed algal cells, the sedimentation rate of the cells was measured in a magnetic field. Four milliliters of the algal suspension was measured into a special cuvette with a 10 × 10 × 3 mm N45 neodymium magnet affixed to the bottom. The cuvette containing the magnet was then placed within the spectrophotometer cuvette holder, and the sedimentation rate was measured by the change in optical density over time. The change in optical density was monitored by measuring at 680 nm using a UV/VIS double-beam spectrophotometer (VWR International, Avantor, Radnor, PA, USA; model PV4).

The harvesting efficiency (HE%) of the process was calculated from the following equation:

$$\mathrm{HE}\%=\frac{{OD}_{0}-{OD}_{1}}{{OD}_{0}}\times 100$$

*OD*_0_ is the initial absorbance of microalgae cultivation at a wavelength of 680 nm.

*OD*_1_ is the absorbance at the same wavelength of the supernatant liquid that separates from the microalgae-particles flocs after the application of the magnetic field.

### 2.5. Characterization Technics

Characterization of the particles size and morphology of the zinc ferrite nanoparticles was carried by high-resolution transmission electron microscopy (HRTEM), Talos F200X G2 electron microscope (Thermo Fisher Scientific, Eindhoven, 5651 GZ, The Netherlands) with field emission electron gun, X-FEG; accelerating voltage of 20–200 kV was used. The imaging and selected area electron diffraction (SAED) measurements were carried out with SmartCam digital search camera (Ceta 16 Mpixel, 4k × 4k CMOS camera) and a high-angle annular dark-field (HAADF) detector (Thermo Fisher Scientific, Eindhoven, 5651 GZ, The Netherlands). For HRTEM examination, the aqueous dispersion of zinc ferrite particles was dropped onto 300 mesh copper grids (Ted Pella Inc., 4595 Redding, CA 96003, USA).

The specific surface area (SSA) measurements of the samples were carried out by nitrogen adsorption–desorption method at 77 K. For this, the Micromeritics ASAP 2020 (Micromeritics Instrument Corp., Norcross, GA 30093, USA) equipment was used, and the evaluation was carried based on the Brunauer–Emmett–Teller (BET) method.

X-ray diffraction measurements (XRD) were carried out by Bruker D8 diffractometer (Cu-Kα source) in parallel beam geometry (Göbel mirror) with Vantec detector (Bruker AXS GmbH, Karlsruhe, D-76187, Germany) due the phase analysis of the zinc ferrite samples. Average crystallite size of the zinc ferrite crystallites was determined by the mean column length calibrated method using the full width at half maximum (FWHM) and the width of the Lorentzian component of the fitted profiles.

Bruker Vertex 70 infrared spectroscope (FTIR) (Bruker AXS GmbH, Karlsruhe, D-76187, Germany) was used for identification of the functional groups of the magnetic nanoparticles. For the measurements, 10 mg zinc ferrite was pelletized with 250 mg potassium bromide; spectra were recorded in transmission mode.

The hydrodynamic diameter of the zinc ferrite particles was measured in an aqueous phase at a pH of 6. This measurement was conducted using a Litesizer 500 equipment manufactured by Anton Paar, a company based in Hamburg, Germany. The dynamic light scattering (DLS) measurements were conducted at room temperature (25 °C) in automatic mode (for backscatter detector fixed at 175°) using a 633 nm He-Ne laser. Samples were measured in polystyrene disposable cuvettes. These cuvettes were manufactured by Anton Paar, a company based in Hamburg, Germany. Zeta potential measurements were carried out based on electrophoretic mobility measurements by applying laser Doppler electrophoresis with a Litesizer 500 equipment (Anton Paar GmbH, A-8054 Graz, Austria). The calculation of zeta potential was carried out by using Smoluchowski equation. The measurements were conducted within an Omega Zeta cell (Mat.No. 225288, Anton Paar GmbH, A-8054 Graz, Austria).

The magnetic behavior of the zinc ferrite samples was studied with self-developed vibrating-sample magnetometer (VSM) system based on a water-cooled Weiss-type electromagnet. For the VSM tests, 20 mg of the sample was pelletized and its magnetization (M) was measured as a function of magnetic field (H) up to 10,000 Oe field strength at room temperature.

The pH of algae cultures was monitored using a VOLTCRAFT PHT-700 pH meter. Microalgal growth was followed by measuring optical density at 680 nm using a UV-6300 double-beam spectrophotometer (VWR International). During the magnetic separation measurements, the change in optical density of the algae dispersion was followed by measuring at 680 nm by UV/VIS double-beam spectrophotometer (VWR International, Avantor, Radnor, PA, USA; model PV4).

Dissolved cadmium concentrations were analyzed using a Varian 720-ES ICP-AES spectrometer operating at 1050 W RF power. Measurements were performed using axial plasma observation. The measurements were performed in triplicate (threefold reading) with an integration time of 10 s. A Sturman-Masters spray chamber and a V-groove nebulizer were utilized for sample introduction. The sample uptake rate was maintained at 2.1 cm^3^ min^−1^. Single-element standards (Merck, Certipur, c: 1000 mg L^−1^) were used for measurement of Cd. The limit of detection (LOD) was 0.05 mg L^−1^.

## 3. Results and Discussion

### 3.1. Formation Mechanism of the Amine-Functionalized Zinc Ferrite Nanoparticles

The proposed formation mechanism of the zinc ferrite particles is assisted by the transformation of MEA, because the presence of zinc(II) ions reduces the thermal decomposition temperature of MEA, releasing ammonia [22]. Furthermore, Rochell has shown that the presence of Fe(III) ions promotes the oxidative degradation of MEA. During this process, an aminium radical is formed, which is deprotonated to an imine radical that is further degraded to ammonia and aldehyde (hydroxyacetaldehyde) via imine [23]. The alkaline conditions due to the released ammonia lead to the formation of Fe(OH)_3_ and Zn(OH)_2_, which can be transformed into ZnFe_2_O_4_ through dehydration.

$${NH}_{2}{CH}_{2}{CH}_{2}OH\overset{{Fe}^{3+}and{-H}^{+}}{\to }H_{2}N^{*}{=CH-CH}_{2}-OH+H_{2}O\overset{{Fe}^{3+}and{-H}^{+}}{\to }HO{CH}_{2}CHO+{NH}_{3}$$

$${NH}_{3}+H_{2}O\to {NH}_{4}^{+}+{OH}^{-}$$

$${Fe}^{3+}+3\;{OH}^{-}\to {Fe(OH)}_{3}$$

$${Zn}^{2+}+2\;{OH}^{-}\to {Zn(OH)}_{2}$$

$${Zn(OH)}_{2}+2\;{Fe(OH)}_{3}\to {ZnFe}_{2}O_{4}+4\;H_{2}O$$

### 3.2. Characterization of the Zinc Ferrite Samples

**Figure 1:**
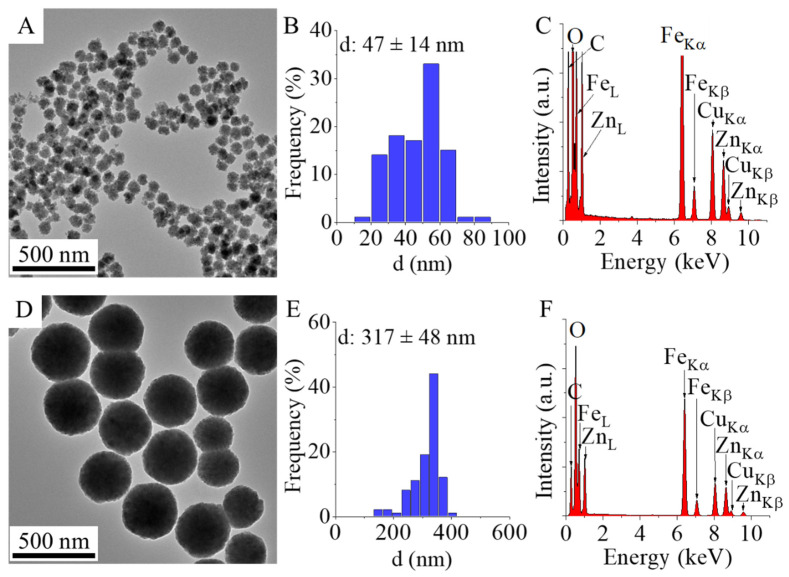
TEM picture, size distribution histogram and EDS spectrum of the ZnFe_2_O_4_ Refl. (**A**–**C**) and ZnFe_2_O_4_ ST. (**D**–**F**) sample.

The specific surface area of the two zinc ferrite samples was measured using the BET method; values of 54 m^2^ g^−1^ for the ZnFe_2_O_4_ ST. sample and 37.5 m^2^ g^−1^ for the ZnFe_2_O_4_ Refl. sample were obtained. High resolution transmission electron microscopic (HRTEM) measurements were carried out for characterization of particle size distribution and the morphology of the two zinc ferrite samples. On the HRTEM images, spherical amine-functionalized zinc ferrite particles are visible with different particle sizes; the ZnFe_2_O_4_ Refl. particles are smaller (d: 47 ± 14 nm) than the particles of the ZnFe_2_O_4_ ST. (317 ± 48 nm) (Figure 1A,B,D,E). It is expected that the significant difference in particle size between the two ferrite samples will cause a significant shift in the magnetization properties as well as in the magnetic separation rate.

On the EDS spectrum are found those elements which make up zinc ferrite, which are zinc, iron and oxygen (Figure 1C,F). The presence of carbon can be explained by the presence of organic compounds adsorbed on the surface of the particles left over from the synthesis (MEA and EG). The carbon peak may come from the material of the grid in TEM studies and is also the cause of the presence of copper (Ted Pella copper grid with carbon layer). The elemental analysis results are presented in Table 1; by comparing the iron-to-zinc ratio, it can be concluded that the ferrites formed during the reaction did not possess a stoichiometric composition. Similar deviations from stoichiometry have been widely reported in the literature for coprecipitated ferrite nanoparticles [24,25,26].

**Figure 2:**
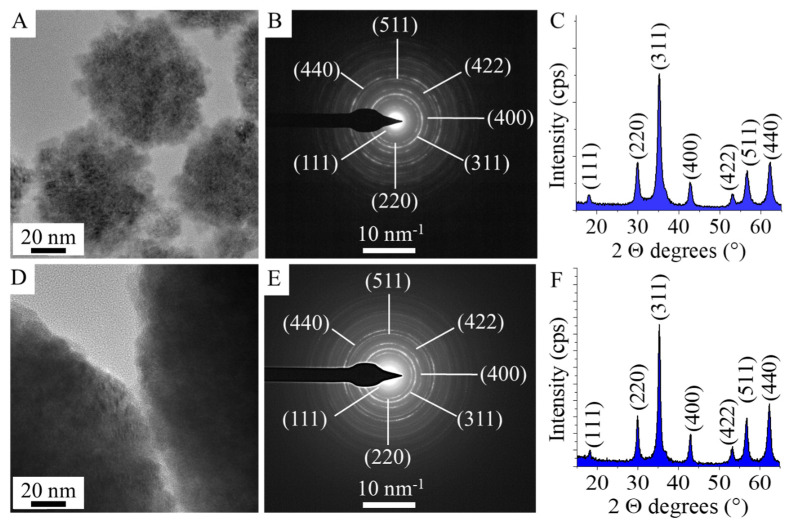
TEM picture, SAED image with the Miller indices and XRD pattern of the ZnFe_2_O_4_ Refl. (**A**–**C**) and ZnFe_2_O_4_ ST. (**D**–**F**) samples.

Selected area electron diffraction (SAED) method was applied to identify the individual particles during the TEM examination (Figure 2A,B,D,E). Correlation was found between d-spacing values of the zinc ferrite spinel and the ring SAED patterns after the evaluation of the SAED images. The lattice plane spacings were determined for zinc ferrite based on an X-ray database (PDF card no. 79-1499). Moreover, XRD measurements were carried out to confirm that no other crystalline phases (iron oxide or zinc oxide) next to zinc ferrite were present in the sample (Figure 2C,F). Only reflections characteristic of zinc ferrite spinel were identified on the XRD diffractograms in the case of two samples. The XRD patterns of the two samples revealed peaks of only ZnFe_2_O_4_, located at (2Th/hkl) 18.1°/(111), 30.0°/(220), 35.2°/(311), 42.8°/(400), 53.1°/(422), 56.6°/(511) and 62.3°/(440) (PDF card no. 79–1499). Other phases as residue were not detected in the two samples; therefore, it can be stated that the two synthesis methods are usable for the preparation of pure spinel phase ZnFe_2_O_4_ nanoparticles.

**Figure 3:**
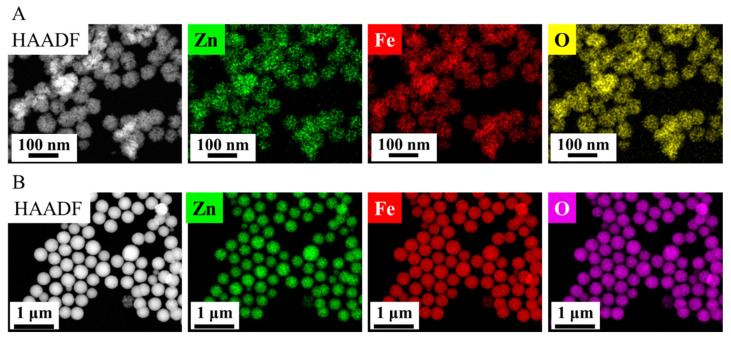
HAADF pictures and element maps of the ZnFe_2_O_4_ Refl. (**A**) and ZnFe_2_O_4_ ST. (**B**) samples.

In the high-angle annular dark-field (HAADF) pictures of the two ferrite samples, the ZnFe_2_O_4_ particles are visible in sharp contrast (Figure 3A,B). Elemental maps were made in the case of two zinc ferrite samples, to see how the different types of elements are positioned in relation to each other (Figure 3A,B). This suggests that zinc, iron and oxygen are present in the zinc ferrite phase in the sample. Based on this, it can be concluded that the particles in the sample are found as zinc ferrite and that no other oxides (e.g., maghemite, hematite, magnetite or zinc oxide) were formed. XRD and SAED studies were also carried out to provide credible evidence for this (Figure 2B–E).

In the synthesis of zinc ferrite, monoethanol was used, which leads to the formation of -NH_2_ functional groups on the surface of the nanoparticles, and FTIR measurements were performed to investigate their presence. Two characteristic bands were found at wavenumbers of 428 cm^−1^ and 577 cm^−1^ in the FTIR spectra of the two ZnFe_2_O_4_ samples; these bands were assigned to the intrinsic stretching vibration modes of the metal–oxygen bonds at the octahedral and tetrahedral sites in the spinel structure [26]. Additionally, a band at 897 cm^−1^ was identified as the rocking vibration mode of the -CH_2_ groups, originating from the adsorbed ethylene glycol and monoethanolamine molecules on the surface of the ferrites [27]. The stretching vibration modes of the C-O and C-N bonds show absorption in the wavenumber range 1000 to 1100 cm^−1^; these belong to the hydroxyl and amine functional groups. Two other absorption bands are identified at 1382 cm^−1^ and 1585 cm^−1^, which are characteristic of the bending vibration mode of -OH functional groups and the νC=C vibration of the adsorbed EG and MEA molecules. The band at 1629 cm^−1^ indicated the presence of -NH_2_ on the surface of zinc ferrite nanoparticles (from the MEA molecules). In convolution with the C=C band is the absorption band of the -OH bending vibration from adsorbed water, which is found at 1650 cm^−1^ [28]. The bands of symmetric and asymmetric stretching vibration of the C-H bonds are shown at 2859 cm^−1^ and 2919 cm^−1^, which belong to the adsorbed EG and MEA on the surface of ZnFe_2_O_4_. The stretching vibration band of the N–H bonds is convoluted with the vibration band of -OH groups in a broad band in the 3000–3750 cm^−1^ region.

**Figure 4:**
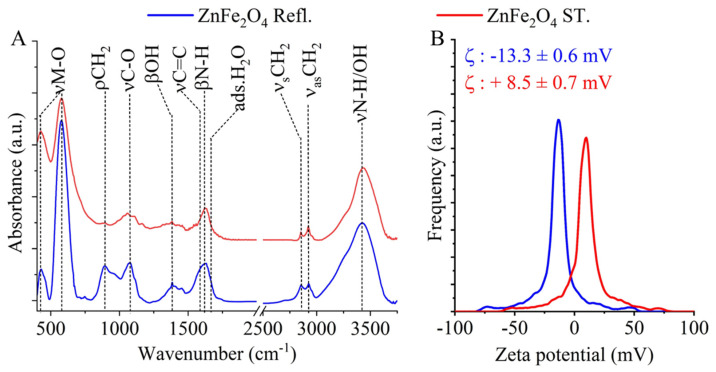
FTIR spectra (**A**) and zeta potential distribution (**B**) of the two zinc ferrite samples.

In the case of zeta potential, a significant difference was measured, because the electrokinetic potential of the ZnFe_2_O_4_ Refl. sample was −13.3 ± 0.6 mV, which is more negative than that of the ZnFe_2_O_4_ ST. sample, where + 8.5 ± 0.7 mV was obtained (Figure 4B). The zeta potential of the *C. vulgaris* algae is −29.0 ± 0.3 mV at pH 6, and thus we can expect that ZnFe_2_O_4_ nanoparticles with positive surface charge will bind more strongly to the algal wing due to electrostatic interaction.

**Figure 5:**
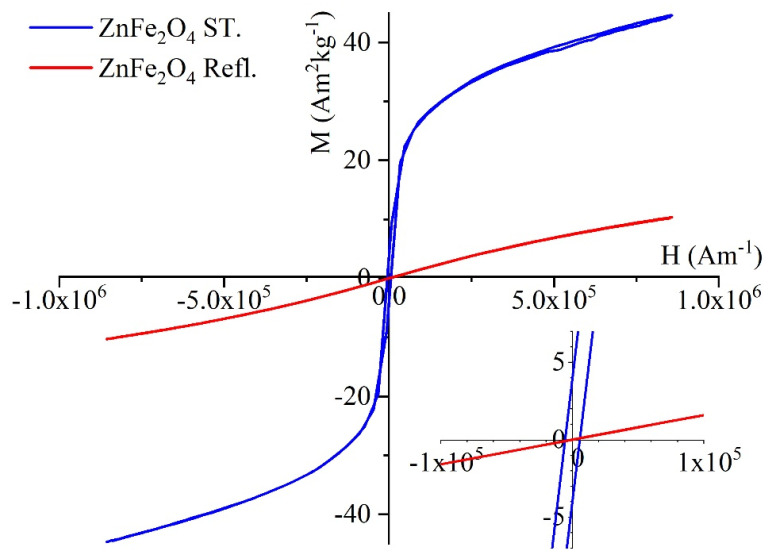
VSM curve of the ZnFe_2_O_4_ ST. (blue line) and ZnFe_2_O_4_ Refl. (red line).

The magnetization curves of the two ZnFe_2_O_4_ samples were made at 298 K using a vibrating-sample magnetometer (VSM) (Figure 5). In the magnetic saturation (Ms) values of the two samples, significant difference was showed, because in the case of ZnFe_2_O_4_ ST. nanoparticles the Ms value was 43 Am^2^kg^−1^ (at 8 × 10^5^ Am^−1^), which is a multiple of the value measured for the ZnFe_2_O_4_ Refl. sample (Ms: 10 Am^2^kg^−1^). As we know, the saturation magnetization value decreases with decreasing particle size due to the spin disorder at the surface of the nanoparticles, which leads to the momentous change in the Ms values at small dimensions [29]. This explains the significant difference in Ms values measured for two zinc ferrite samples, for which there are significant differences in particle sizes (47 ± 14 nm and 317 ± 48 nm). The magnetization curves of the ZnFe_2_O_4_ Refl. sample do not exhibit a hysteresis loop, and in this sense the values of the remanent magnetization (Mr) and coercivity (Hc) were approximately zero. It can be stated that the nanoparticles of the ZnFe_2_O_4_ Refl. sample exhibit superparamagnetic behavior at room temperature. In contrast, the VSM curve of the ZnFe_2_O_4_ ST. sample shows hysteresis, the values of Hc (5.3 × 10^3^ Am^−1^) and Mr (3.7 Am^2^kg^−1^) confirming a ferromagnetic property.

Several synthesis methods are found in the international literature which resulted in zinc ferrite nanoparticles with similar morphology as the experimental conditions we used [30,31,32,33,34]. Magnetic properties are varied; it is possible to produce both superparamagnetic and soft ferromagnetic nanoparticles using this method, with saturation magnetization ranging from 43 to 81 Am^2^kg^−1^, residual magnetization from 0 to 8 Am^2^kg^−1^ and coercivity varying within the range 0–8000 Am^−1^, depending on the reaction conditions (Table 2). Based on the literature data, spherical aggregates range from 10 to 345 nm, whereas the average sizes of the particles we synthesized are 47 ± 17 and 317 ± 78 nm (Table 2).

**Figure 6:**
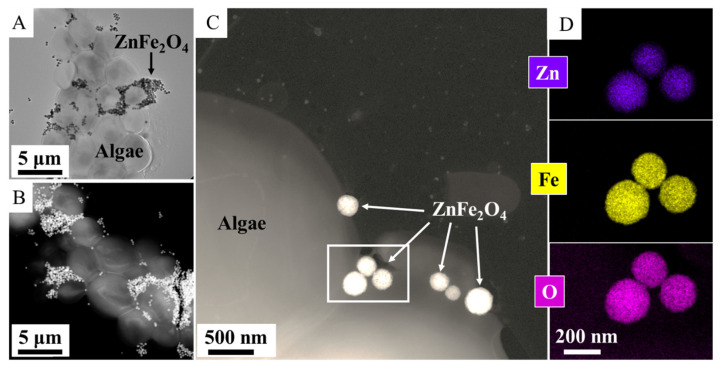
TEM (**A**), HAADF pictures (**B**,**C**), element maps (**D**) of the zinc ferrite nanoparticles-treated algae after magnetic separation.

To confirm the presence of magnetic nanoparticles on the surface of the algae, electron microscopy was used (Figure 6A). The high-angle annular dark-field (HAADF) images show the zinc ferrite particles with a brighter contrast on the surface of the algae (Figure 6B,C). The elemental maps show the position of zinc, iron and oxygen, indicating the positions of the ZnFe_2_O_4_ nanoparticles (Figure 6D). Furthermore, the zinc ferrite particles were also well bound on the surface of the algae, and therefore, by applying a magnetic field, the algae could be effectively recovered from the purified water sample.

### 3.3. Results of the Cadmium Adsorption Tests by Using Chlorella vulgaris

To evaluate the interplay between metal toxicity and biological metal uptake, a combined growth inhibition and removal kinetic assay was performed. Unlike static adsorption studies using constant adsorbent masses, this experiment employed actively growing *C. vulgaris* cultures to monitor how cadmium exposure simultaneously affects microalgal proliferation and how the developing biomass contributes to the dynamic depletion of dissolved metal ions over time.

**Figure 7:**
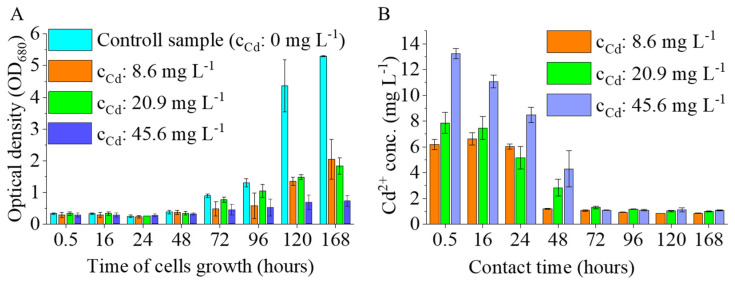
Effect of Cd^2+^ ions on *C. vulgaris* growth (OD_680_) (**A**) and cadmium removal efficiency vs. contact time (**B**) at 8.6, 20.9, and 45.6 mg L^−1^ cadmium concentrations.

The effect of cadmium on the growth of *Chlorella vulgaris* was evaluated by monitoring changes in optical density at 680 nm (OD_680_) over time under control conditions and in the presence of 8.6, 20.9, and 45.6 mg L^−1^ dissolved cadmium (Figure 7A).

In control cultures, *C. vulgaris* exhibited sustained growth following a short adaptation phase, with OD_680_ increasing markedly after 72 h and reaching values of approximately 8.0 at 168 h. In contrast, cadmium exposure resulted in pronounced growth inhibition at all tested concentrations. Compared to the control, OD_680_ values in cadmium-treated cultures remained substantially lower throughout the experimental period.

At 168 h, growth inhibition relative to the control culture exceeded 60% at 8.6 mg L^−1^ Cd and reached approximately 75% and 70% at 20.9 and 45.6 mg L^−1^ Cd, respectively, based on OD_680_ values. Notably, growth suppression was already evident during the early cultivation phase and persisted throughout the experiment.

Within the applied cadmium concentration range, no clear dose-dependent trend in growth inhibition was observed. All tested cadmium levels caused severe suppression of microalgal growth, resulting in similarly low OD_680_ values, particularly at later time points. This indicates that even the lowest applied cadmium concentration was sufficient to strongly inhibit *C. vulgaris* proliferation under experimental conditions.

The temporal evolution of dissolved cadmium concentrations in *Chlorella vulgaris* cultures exposed to nominal cadmium levels of 8.6, 20.9, and 45.6 mg L^−1^ is shown in Figure 7B. Initial dissolved cadmium concentrations were determined immediately after cadmium addition and prior to inoculation (0 h), providing the reference levels for subsequent removal.

Following inoculation, a rapid decrease in dissolved cadmium concentration was observed for all treatments. Already at the first post-inoculation sampling point (0.5 h), dissolved cadmium concentrations were lower than the corresponding initial values, indicating fast removal from the aqueous phase. This initial decrease was evident across all cadmium levels and was most pronounced at the highest nominal concentration (45.6 mg L^−1^).

Between 0.5 and 24 h, dissolved cadmium concentrations continued to decline substantially, reflecting an intense early removal phase. The rate of cadmium decreases during this period exhibited a clear concentration dependence, with higher initial cadmium levels showing larger absolute reductions.

From 24 to 72 h, further decreases in dissolved cadmium concentration were observed for all treatments; however, the rate of decline was markedly lower compared to the initial phase. After 72 h of cultivation, dissolved cadmium concentrations approached low residual levels and showed only minor temporal changes up to the end of the experiment at 148 h.

At later time points (≥72 h), dissolved cadmium concentrations converged toward similarly low values regardless of the initial cadmium level, suggesting stabilization of the aqueous cadmium fraction. Variability among parallel cultures remained limited throughout the experiment, as indicated by the relatively small standard deviations.

Summarizing the above results, it can be concluded that the investigated algal system is highly effective for cadmium removal across a broad concentration range. A significant decrease in cadmium content was observed after only half an hour. After 72 h, values of 1.1 ± 0.06 mg L^−1^, 1.3 ± 0.01 mg L^−1^ and 1.1 ± 0.02 mg L^−1^ were measured for all three initial cadmium concentrations, which showed no further significant decrease even after 168 h. The cadmium removal efficiencies calculated for the three initial concentrations were 89.0% (10 mg L^−1^), 93.5% (20 mg L^−1^), and 97.6% (45 mg L^−1^).

The key finding of our research is the analysis of the impact of cadmium on the viability of *Chlorella vulgaris*. Our results demonstrate that Cd exposure induces a pronounced, non-dose-dependent inhibition of microalgal growth, even at minimal concentrations. This toxicity is attributed to multiple mechanisms, including the disruption of redox homeostasis, oxidative stress induction, and the inactivation of essential enzymes through sulfhydryl group binding. Despite severe suppression of biomass accumulation, Cd removal from the aqueous phase remained highly efficient. This indicates a significant decoupling of metal remediation from algal proliferation. The high removal efficiency is driven by rapid, growth-independent mechanisms: surface-associated adsorption (via carboxyl, phosphate, and hydroxyl groups) and energy-intensive intracellular sequestra-tion mediated by phytochelatins and metallothioneins. These findings challenge the pre-vailing paradigm that high biomass productivity is a prerequisite for effective heavy metal removal, suggesting that *C. vulgaris* remains a viable candidate for the treatment of highly contaminated wastewater, where metal toxicity limits growth.

### 3.4. Results of Magnetic Separation of Cadmium-Adsorbed Chlorella vulgaris

**Figure 8:**
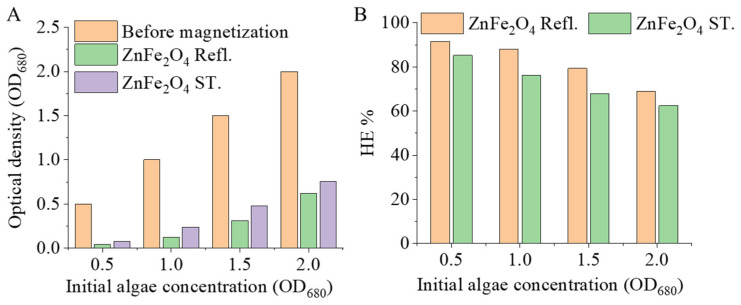
Magnetic separation of Cd^2+^-adsorbed *Chlorella vulgaris* using ZnFe_2_O_4_ nanoparticles. Optical density (OD_680_) of the algal suspensions before magnetization and after magnetic separation in the presence of ZnFe_2_O_4_ Refl. or ZnFe_2_O_4_ ST. at different initial algae concentrations (**A**). Harvesting efficiency (HE%) achieved with ZnFe_2_O_4_ Refl. and ZnFe_2_O_4_ ST. as a function of initial algae concentration (**B**).

After Cd^2+^ adsorption, amine-functionalized zinc ferrite nanoparticles were added to the algal biomass, and then the algae were separated from the suspension using a magnetic field. At low pH values, both types of zinc ferrite nanoparticle exhibited a strong association with Cd^2+^-adsorbed algal cells, resulting in efficient magnetic separation. At the same time, the number of separated algae increased systematically with increasing initial biomass concentration, indicating a gradual decrease in separation efficiency at higher cell densities. For the highest-concentration Cd^2+^-adsorbed algal suspension, OD_680_ decreased from 2.0 to 0.622 with ZnFe_2_O_4_ Refl. and to 0.752 with ZnFe_2_O_4_ ST., corresponding to harvesting efficiencies of 68.9% and 62.4%, respectively. For the lowest-concentration suspension, OD_680_ decreased from 0.5 to 0.042 with ZnFe_2_O_4_ Refl. and to 0.074 with ZnFe_2_O_4_ ST., resulting in harvesting efficiencies of 91.6% and 85.2%, respectively (Figure 8A,B). The decline in HE% observed to be concentration-dependent is consistent with a lower effective nanoparticle to cell ratio and higher solids volume fraction at elevated biomass loadings. This decline can be attributed to a reduction in available adsorption sites, suppression of particle–cell encounter and attachment rates via steric and hydrodynamic hindrance, and a decrease in the magnetophoretic mobility of nanoparticle–cell heteroaggregates. The persistent performance discrepancy between the two zinc ferrite nanoparticle variants suggests disparities in interfacial affinity and heteroaggregation or flocculation propensity, resulting in more readily magnetically recoverable aggregates for ZnFe_2_O_4_ Refl., particularly under increased biomass loading conditions.

**Figure 9:**
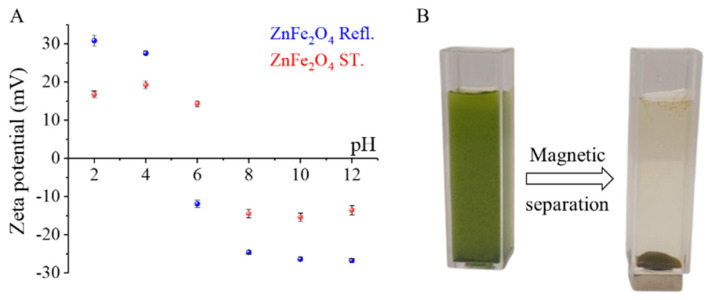
Zeta potential (mV, right axis) measured across the investigated pH range ZnFe_2_O_4_ Refl. and ZnFe_2_O_4_ ST. particles (**A**) and the algal biomass magnetization before and after (**B**).

The pH-dependent zeta potential measurements of the ZnFe_2_O_4_ nanoparticle suspensions indicate that both materials carry a positive surface charge under acidic conditions (pH 2–4), attributable to the protonation of surface amine functionalities in this pH range (Figure 9A). These pH values correspond to the initial pH values to which the algal suspensions were adjusted prior to ZnFe_2_O_4_ addition in the magnetic harvesting experiments. As demonstrated in the relevant literature, the zeta potential of *Chlorella vulgaris* suspensions is reported to be negative at pH values above approximately 2. This finding indicates that deprotonated surface functional groups, primarily carboxylate and phosphate groups, predominate over the net electrokinetic charge of the cell wall. Consequently, positively charged ZnFe_2_O_4_ nanoparticles can interact strongly with the overall negatively charged algal surface via electrostatic attraction within this pH range. In this context, the positive ζ-potential of ZnFe_2_O_4_ nanoparticles at pH 2–4 (~+15 to +20 mV) is expected to reduce the electrostatic energy barrier to contact and encourage nanoparticle to cell heteroaggregation and floc formation, resulting in compact, magnetically recoverable aggregates and explaining the observed high recovery under strongly acidic conditions (Figure 9B).

## 4. Conclusions

This study investigates the toxicological impact and sequestration potential of cadmium (Cd) in *Chlorella vulgaris* cultures, combined with the removal of cadmium-bound biomass via magnetic separation. Using the polyol method, amine-functionalized zinc ferrite nanoparticles were synthesized under two different conditions. The solvothermal (ZnFe_2_O_4_ ST) and atmospheric pressure (ZnFe_2_O_4_ Refl.) synthesis routes significantly influenced the size, morphology, surface charge, and magnetic properties of the nanoparticles. The zeta potential of the ZnFe_2_O_4_ Refl. sample exhibited a much stronger pH dependence compared to its solvothermal counterpart. Consequently, the ZnFe_2_O_4_ Refl. sample proved more effective in the removal of algae through magnetic separation. The integration of zinc ferrite nanoparticles allows for the rapid recovery of Cd-adsorbed algal biomass via magnetic separation. The most effective separation was observed under acidic conditions, where there was a strong association between both nanoparticle types and Cd^2+^-loaded cells. As the biomass concentration increased, the harvesting efficiency decreased. At an initial OD_680_ of 2.0, the OD_680_ fell to HE% values of 68.9% and 62.4%, respectively, whereas at an initial OD_680_ of 0.5, higher HE% values of 91.6% and 85.2% were recorded. This concentration-dependent decline is consistent with a reduced effective nanoparticle to cell ratio and higher solids volume fraction, which can suppress nanoparticle and cell interactions, limit the available binding sites, and reduce the magnetophoretic mobility of nanoparticle–cell heteroaggregates. Across all loadings, ZnFe_2_O_4_ Refl. consistently achieved lower residual OD680 and higher HE% than ZnFe_2_O_4_ ST, indicating stronger interfacial affinity and/or greater flocculation propensity. Zeta potential data demonstrate that both zinc ferrite suspensions possess a positive charge at pH 2–4 (~+15 to +20 mV). Conversely, *C. vulgaris* is reported to exhibit a negative charge above approximately pH 2, attributable to deprotonated carboxylate and phosphate groups. This supports the hypothesis that electrostatically driven heteroaggregation is the primary mechanism for efficient magnetic recovery at a low pH.

The integration of *Chlorella vulgaris* adsorption with amine-functionalized zinc ferrite nanoparticles led to the creation of a highly efficient, synergistically acting system for cadmium remediation. While previous studies have examined zinc ferrite as a direct adsorbent for heavy metals or used other magnetic nanoparticles (such as magnetite) for microalgal harvesting, this study demonstrates for the first time that synthesized ZnFe_2_O_4_ nanoparticles can effectively aggregate and separate cadmium-laden algal biomass without the need for additional chemical flocculants. Furthermore, we provided that the surface charge interactions between the amine-functionalized nanoparticles and the algal cell wall are sufficient to achieve over 90% algae-harvesting efficiency even under significant heavy metal stress.

## Figures and Tables

**Table 1:** Elemental composition of zinc ferrite samples.

Sample ID	Element	Atomic Fraction (%)	Atomic Error (%)	Mass Fraction (%)	Mass Error (%)
ZnFe_2_O_4_ Refl.	Fe	72.98	2.91	69.75	3.12
	Zn	27.02	2.91	30.25	3.12
	O	67.49	4.92	36.67	5.2
ZnFe_2_O_4_ ST.	Fe	77.12	2.61	74.21	2.83
	Zn	22.88	2.61	25.79	2.83
	O	61.02	5.35	30.77	4.80

**Table 2:** Comparison table for magnetic properties and particle size of zinc ferrite nanoparticles synthesized by solvothermal methods.

Synthesis Method	Ms (Am^2^ kg^−1^)	Mr (Am^2^ kg^−1^)	Hc (Am^−1^)	d (nm)	Ref.
Solvothermal	10	0	0	47 ± 17	This work
	43	3.7	5300	317 ± 48	
	60.4	0.83	788	150 ± 25	[30]
	60.3	0.18	16	130 ± 30	
	52	1.31	1751	120 ± 30	
	43.2	0.35	167	300 ± 50	
	66.71	0.42	292	345.2	[31]
	58.46	0.30	251	340.8	
	66.52	0.38	280	312.8	
	66.71	0	0	345.2	[32]
	81.34	5.2	2753	150.6	
	76.65	7.5	4078	110.6	
Solvothermal (reflux)	50.4	0	0	10	[33]
Hydrothermal	10	2	7958	n.d.	[34]

## Data Availability

Data is contained within the article.

## Abbreviations

The following abbreviations are used in this manuscript:

HRTM	High-Resolution Transmission Electron Microscopy
FTIR	Fourier Transform Infrared Spectroscopy
SEM	Scanning Electron Microscopy
VSM	Vibrating-Sample Magnetometry
PDDA	Poly diallyldimethylammonium chloride
PEI	Polyethylenimine
APTES	3-aminopropyl triethoxysilane
SAED	Selected Area Electron Diffraction
HAADF	High-Angle Annular Dark-Field
ICP-AES	Inductively Coupled Plasma Atomic Emission Spectrometry

## References

[1] Vidu R., Matei E., Predescu A.M., Alhalaili B., Pantilimon C., Tarcea C., Predescu C. (2020). Removal of Heavy Metals from Wastewaters: A Challenge from Current Treatment Methods to Nanotechnology Applications. Toxics.

[2] Qasem N.A.A., Mohammed R.H., Lawal D.U. (2021). Removal of heavy metal ions from wastewater: A comprehensive and critical review. npj Clean Water.

[3] Kubier A., Wilkin R.T., Pichler T. (2019). Cadmium in soils and groundwater: A review. Appl. Geochem..

[4] Genchi G., Sinicropi M.S., Lauria G., Carocci A., Catalano A. (2020). The Effects of Cadmium Toxicity. Int. J. Environ. Res. Public Health.

[5] Bouida L., Rafatullah M., Kerrouche A., Qutob M., Alosaimi A.M., Alorfi H.S., Hussein M.A. (2022). A Review on Cadmium and Lead Contamination: Sources, Fate, Mechanism, Health Effects and Remediation Methods. Water.

[6] Khan Z., Elahi A., Bukhari D.A., Rehman A. (2022). Cadmium sources, toxicity, resistance and removal by microorganisms—A potential strategy for cadmium eradication. J. Saudi Chem. Soc..

[7] International Agency for Research on Cancer (IARC) (1993). Cadmium and Cadmium Compounds. Beryllium, Cadmium, Mercury, and Exposures in the Glass Manufacturing Industry.

[8] Ahalya N., Ramachandra T.V., Kanamadi R.D. (2003). Biosorption of Heavy Metals. Res. J. Chem. Environ..

[9] Houessionon M.G.K., Ouendo E.-M.D., Bouland C., Takyi S.A., Kedote N.M., Fayomi B., Fobil J.N., Basu N. (2021). Environmental Heavy Metal Contamination from Electronic Waste (E-Waste) Recycling Activities Worldwide: A Systematic Review from 2005 to 2017. Int. J. Environ. Res. Public Health.

[10] Cheng J., Yin W., Chang Z., Lundholm N., Jiang Z. (2017). Biosorption capacity and kinetics of cadmium(II) on live and dead *Chlorella vulgaris*. J. Appl. Phycol..

[11] Mahlangu D., Moyo W., Popoola A.P.I., Akinlabi E.T. (2024). Microalgae-Mediated Biosorption for Effective Heavy Metal Removal from Water and Wastewater: A Review. Water.

[12] Deepa P., Sowndhararajan K., Kim S. (2023). A Review of the Harvesting Techniques of Microalgae. Water.

[13] Wang S.-K., Stiles A.R., Guo C., Liu C.-Z. (2015). Harvesting microalgae by magnetic separation: A review. Algal Res..

[14] Seo J.Y., Jeon H.J., Kim J.W., Lee J., Oh Y.K., Ahn C.W., Lee J.W. (2018). Simulated-Sunlight-Driven Cell Lysis of Magnetophoretically Separated Microalgae Using ZnFe_2_O_4_ Octahedrons. Ind. Eng. Chem. Res..

[15] Sonu, Sharma S., Dutta V., Raizada P., Hosseini-Bandegharaei A., Thakur V., Nguyen V.-H., VanLe Q., Singh P. (2021). An overview of heterojunctioned ZnFe_2_O_4_ photocatalyst for enhanced oxidative water purification. J. Environ. Chem. Eng..

[16] Li L., Wang J., Fang H. (2025). Fabrication of ZnFe_2_O_4_@g-C_3_N_4_ for enhanced photo-Fenton effect and visible light-driven organic dye degradation. Sci. Rep..

[17] Rasheed-Adeleke A.A., Olatunde O.C., Seheri N.H., Oyewo O.A., Ferjani H., Onwudiwe D.C. (2025). Synthesis and photocatalytic performance of ZnFe_2_O_4_ on the degradation of tetracycline in water. Appl. Phys. A.

[18] Palusamy S., Inbasekaran M., Anbazhagan S., Balu K., Durai M., Ganesamoorthy T., Ravichandran S., Alam P., Durai M., Ahn Y.-H. (2025). Pd-TiO_2_/ZnFe_2_O_4_ photocatalyst for photocatalytic degradation of ciprofloxacin under solar light irradiation. J. Mol. Struct..

[19] Madhusudan P., Lee C., Kim J.-O. (2024). Hierarchical ZnO/ZnFe_2_O_4_ yolk-shell adsorbent as a promising material for phosphate recovery and adsorption of organic pollutants from the simulated wastewater. Sep. Purif. Technol..

[20] Al-Najar B., Modwi A., Shaikh M.N., Bououdina M., Albuflasa H., Hankins N.P. (2024). Highly nanocrystalline Mg doped ZnFe_2_O_4_ powders for rapid and simultaneous adsorption of lead, copper, and cadmium heavy metals ions in synthetic/sea waters. J. Alloys Compd..

[21] Xu Q., Hou G., Chen J., Wang H., Yuan L., Han D., Hu Q., Jin H. (2021). Heterotrophically Ultrahigh-Cell-Density Cultivation of a High Protein-Yielding Unicellular Alga Chlorella With a Novel Nitrogen-Supply Strategy. Front. Bioeng. Biotechnol..

[22] Wei M., Huang A.-C., Shu C.-M., Zhang L. (2019). Thermal Decomposition and Nonisothermal Kinetics of Monoethanolamine Mixed with Various Metal Ions. Sci. Rep..

[23] Makovec D., Drofenik M. (2008). Non-Stoichiometric Zinc-Ferrite Spinel Nanoparticles. J. Nanopart. Res..

[24] Ammar S., Jouini N., Fievet F., Beji Z., Smiri L.S., Molinie P. (2004). Influence of the Synthesis Parameters on the Cationic Distribution of ZnFe_2_O_4_ Nanoparticles Obtained by Forced Hydrolysis in Polyol Medium. J. Non-Cryst. Solids.

[25] Sutka A., Mezinskis G., Lusis A., Jakovlevs D. (2012). Influence of Iron Non-Stoichiometry on Spinel Zinc Ferrite Gas Sensing Properties. Sens. Actuators B Chem..

[26] Chi S., Rochelle G.T. (2002). Oxidative Degradation of Monoethanolamine. Ind. Eng. Chem. Res..

[27] Ikramullah, Ali N., Ali F., Sheikh Z.A., Bilal M., Ahmad I. (2020). Photocatalytic Performance of Zinc Ferrite Magnetic Nanostructures for Efficient Eriochrome Black-T Degradation from the Aqueous Environment under Unfiltered Sunlight. Water Air Soil Pollut..

[28] Cui L., Guo P., Zhang G., Li Q., Wang R., Zhou M., Ran L., Zhao X.S. (2013). Facile synthesis of cobalt ferrite submicrospheres with tunable magnetic and electrocatalytic properties. Colloids Surf. A Physicochem. Eng. Asp..

[29] Caizer C., Aliofkhazraei M. (2016). Nanoparticle Size Effect on Some Magnetic Properties. Handbook of Nanoparticles.

[30] Guo P., Lv M., Han G., Wen C., Wang Q., Li H., Zhao X. (2016). Solvothermal Synthesis of Hierarchical Colloidal Nanocrystal Assemblies of ZnFe_2_O_4_ and Their Application in Water Treatment. Materials.

[31] Shaterian M., Rezvani A., Abbasian A.R. (2019). Controlled synthesis and self-assembly of ZnFe_2_O_4_ nanoparticles into microspheres by solvothermal method. Mater. Res. Express.

[32] Shaterian M., Rezvani A., Abbasian A.R. (2021). Controllable synthesis of ZnFe_2_O_4_ sub-microparticles by poly(diallyldimethylammonium chloride)-assisted solvothermal method. J. Polym. Res..

[33] Manohar A., Krishnamoorthi C., Naidu K.C.B., Pavithra C. (2019). Dielectric, magnetic hyperthermia, and photocatalytic properties of ZnFe_2_O_4_ nanoparticles synthesized by solvothermal reflux method. Appl. Phys. A.

[34] Golsefidi M.A., Abrodi M., Abbasi Z., Dashtbozorg A., Rostami M.E., Ebadi M. (2016). Hydrothermal method for synthesizing ZnFe_2_O_4_ nanoparticles, photo-degradation of Rhodamine B by ZnFe_2_O_4_ and thermal stable PS-based nanocomposite. J. Mater. Sci. Mater. Electron..

